# Integrating mental and physical health care: lessons from the Second World Congress on Integrated Care in Sydney

**DOI:** 10.5334/ijic.2011

**Published:** 2015-03-10

**Authors:** David Perkins

**Affiliations:** Editorial Board, International Journal of Integrated Care

On November 23–25th 2014, the International Foundation for Integrated Care and the Centre for Rural and Remote Mental Health, University of Newcastle, New South Wales, hosted the Second World Congress on Integrated Care in Sydney, Australia [1, 2]. A key theme of the conference was the integration of physical and mental health care. This is a topic which is replete with memorable slogans such as “no health without mental health”, “the contributing life”, “parity for mental health care” and the simple statement “living well”. Each suggests in some way that people with mental health problems may not be treated well in health and social care systems when compared with those with equally debilitating physical symptoms. The phrase “Social and Emotional Wellbeing” is used by Aboriginal and Indigenous peoples and implies a sense of harmony with oneself, ones community and indeed the land. This is much more that the control or absence of symptoms.

Key speakers included the researcher and policy analyst Chris Naylor from the Kings Fund, Brenda Reiss Brennan from Intermountain Health Care in the USA, Rahul Shidhaye from the Public Health Foundation of India and John Feneley, Mental Health Commissioner of New South Wales. Numerous papers in parallel sessions addressed service developments for young people, adults, those with enduring diseases and sufferers from dementia.

Hernan Montenegro set the scene by describing the World Health Organization strategy on patient-centred and integrated care. World Health Organization strategies face many hurdles. They must be internationally relevant and not simply options for rich or indeed poor countries. They must also meet the requirement of contributing to health defined as more than the absence of disease. They must be capable of implementation and scaling up to meet population needs not tailored for small idiosyncratic populations in communities with exceptional leadership, vision and capabilities. The combination of patient centric and integrated care does not allow the adoption of a purely provider driven perspective which is a key cause of fragmented services.

The case for patient-centred and integrated care presented so powerfully at the Congress could be said to have four elements: humanitarian, clinical, economic and political.

The humanitarian case starts with the treatment gap which is widely reported but consistent. People with mental health conditions go untreated at a rate that would be unacceptable for other diseases and when they access care their physical symptoms are often overshadowed and untreated. Many with enduring mental health conditions report that the elapsed time between the appearance of symptoms and receipt of a diagnosis exceeds 10 years. Thus people with mental health conditions bear a disproportionate share of the burden of disease and life expectancies can be reduced by between 10 and 20 years. Causes of death are predominantly the associated chronic and enduring physical diseases.

The clinical case is based around the pattern and severity of mental and physical health comorbidities. The presence of a mental health co-morbidity can have a dramatic impact on symptoms from a physical condition. For instance, patients with cardiovascular disease who also have depression suffer 50% more exacerbations than those without [3]. Clinical outcomes improve when the full range of symptoms are addressed by competent teams of providers working with patients and carers [4].

The economic case centres around evidence presented on the benefits of treating people with mental and physical conditions promptly, with access to appropriate teams of providers, peers and carers, in primary care or community settings. This is in marked contrast to reliance on activity-based or “industrial” funding models that prioritise procedures over outcomes and distort provider behaviour. These benefits have yet to be realised at health system level but there are enough service evaluations to suggest that economic benefits could be realised more widely.

The participation of the NSW Mental Health Commissioner in the Congress highlighted two elements of the political case. Extensive consultations with individuals, communities and carers demonstrate that patients, friends and families are interested in a broad range of outcomes including meaningful social participation, education and employment, secure housing and incomes, as well as improvement in symptoms. These aspirations challenge governments since they may appear to be both unaffordable and unachievable. As Mental Health Commissions are required to report independently on the broad performance of health systems, it will be interesting to see whether public demands for patient-centred and integrated mental health care grow and bear fruit.

These four elements of the case for integrated mental and physical care presented by international experts appear self-evident but are still far from received wisdom in the design and development of health systems. In the first place they challenge years of health system practice in which acute care providers congregated in the hospital and mental health services were provided in distant asylums. This separation is exacerbated by the status differentials within the medical profession in which procedularists lead and psychiatrists bring up the rear. These differentials are replicated in the medical and nursing curricula and so the separation of mind and body is reinforced. The place of primary mental health care has yet to be consolidated within general practice which is the first point of contact in most systems.

At a policy level similar fractures can be identified in policy and governmental systems which further complicate the provision of patient-centred and integrated health and social care. The separation of powers implicit in government structures from national or federal to local encourages cost shifting and blame games and few systems are willing to let clients purchase their own care.

The Congress generated a wealth of evidence which is freely available by consulting the Congress website at http://www.integratedcarefoundation.org/conference/2_world and the Congress supplement of abstracts at http://www.ijic.org/index.php/ijic/issue/view/92 which has contact details for lead authors. It is intended that the impact of the conference should go far beyond the experiences of those who were able to attend the conference in person.

In conclusion, it is hard to understate the importance of the integration of acute, mental health and social services but there is much work to be done. Changes are needed in client services, service models and structures, funding and incentive mechanisms, education and training, national and international health policies. The IJIC welcomes papers that address each of these aspects of patient centres and integrated mental health care. Describing the problem will not suffice, solutions are needed from clients, practitioners, managers and policy-makers. This edition of the IJIC includes a challenging viewpoint from Prof Sharon Lawn which addresses participation in mental health care by service users as a pathway to patient-centred and integrated care [5]. We need more evidence about interventions that produce improved outcomes and the contexts in which they can be successful.

## Note

The International Conference on Integrated Care will be held in Edinburgh, Scotland on March 25–27. The conference website is http://www.integratedcarefoundation.org/conference/15_annual
